# Effect of Photon Hormesis on Dose Responses to Alpha Particles in Zebrafish Embryos

**DOI:** 10.3390/ijms18020385

**Published:** 2017-02-11

**Authors:** Candy Yuen Ping Ng, Shuk Han Cheng, Kwan Ngok Yu

**Affiliations:** 1 Department of Physics and Materials Science, City University of Hong Kong, Hong Kong, China; yuenpng3-c@my.cityu.edu.hk; 2 Department of Biomedical Sciences, City University of Hong Kong, Hong Kong, China; 3 State Key Laboratory in Marine Pollution, City University of Hong Kong, Hong Kong, China

**Keywords:** zebrafish embryos, ionizing radiation, photon hormesis

## Abstract

Photon hormesis refers to the phenomenon where the biological effect of ionizing radiation with a high linear energy transfer (LET) value is diminished by photons with a low LET value. The present paper studied the effect of photon hormesis from X-rays on dose responses to alpha particles using embryos of the zebrafish (*Danio rerio*) as the in vivo vertebrate model. The toxicity of these ionizing radiations in the zebrafish embryos was assessed using the apoptotic counts at 20, 24, or 30 h post fertilization (hpf) revealed through acridine orange (AO) staining. For alpha-particle doses ≥ 4.4 mGy, the additional X-ray dose of 10 mGy significantly reduced the number of apoptotic cells at 24 hpf, which proved the presence of photon hormesis. Smaller alpha-particle doses might not have inflicted sufficient aggregate damages to trigger photon hormesis. The time gap *T* between the X-ray (10 mGy) and alpha-particle (4.4 mGy) exposures was also studied. Photon hormesis was present when *T* ≤ 30 min, but was absent when *T* = 60 min, at which time repair of damage induced by alpha particles would have completed to prevent their interactions with those induced by X-rays. Finally, the drop in the apoptotic counts at 24 hpf due to photon hormesis was explained by bringing the apoptotic events earlier to 20 hpf, which strongly supported the removal of aberrant cells through apoptosis as an underlying mechanism for photon hormesis.

## 1. Introduction

Linear energy transfer (LET) describes the quality of an ionizing radiation. In general, high-LET radiation, such as alpha particles and heavy ions, generate clusters of damage along their trajectories in a medium, while low-LET radiation, such as X-rays and gamma rays, mainly induce dispersed damage, so high-LET radiation can induce more severe biological effects in cells [1]. It was also established that high- and low-LET radiation damage had different kinetics of induction and repair [2,3].

In fact, all living organisms, including humans, are commonly exposed to more than a single type of radiation in our environment. For instance, the general public is exposed to background gamma radiation together with alpha-particle exposures from indoor radon levels [4,5,6,7,8,9,10]. Airline crew members and astronauts are exposed to cosmic radiation and secondary radiation, which consists of neutrons and gamma rays, produced by interaction of the cosmic rays with the shielding materials of the cabin during airplane and space flights [11,12]. Cancer patients could also be exposed to mixed beams of high- and low-LET radiation during radiotherapy. In boron neutron capture therapy, patients are exposed to radiation fields consisting of a mixture of radiation with different LETs, including the high-LET products generated during the ^10^B(n,α)^7^Li reaction and the low-LET gamma rays released during radioactive capture (n,γ) reactions [13].

The biological effects of simultaneous or sequential exposures to high- and low-LET radiations have been studied for more than 40 years. Durand and Olive [14] explored, but did not observe, the repair of sub-lethal damage of combinations of X-rays and neutrons in V79 cells. In contrast, Ngo and Elkind [15] demonstrated the repair of sub-lethal damages of a neutron dose followed by an X-ray dose in V79 cells. McNally et al. [16] and Ngo et al. [17] studied the biological effects of X-rays and neutrons/heavy ions, and the data appeared to imply that X-rays and alpha particles would also interact synergistically to increase cell killing and mutation [18,19]. In contrast, Barendsen et al. [2] reported that the damage from X-rays and alpha particles interacted independently. Brooks et al. [20] showed that in lung epithelial cells, X-rays and alpha particles did not interact to change the cell cycle progression, but interacted to enhance cell killing and micronucleus induction.

More recently, Rithidech and Scott [21] reported an interesting gamma-ray hormetic effect where the biological effect of neutrons was alleviated by a simultaneous gamma-ray exposure. Hormetic responses are generally characterized by biphasic dose-response relationships showing low-dose stimulation and high-dose inhibition [22,23,24,25,26]. Rithidech and Scott [21] confirmed the presence of gamma-ray hormesis in low-dose neutron irradiation through the induction of micronucleated cells in human lymphocytes which had been irradiated with mono-energetic neutron sources with five different energies (i.e., 0.22, 0.44, 1.5, 5.9, and 13.7 MeV) and with 1%, 1%, 2%, 6%, and 6% gamma-ray contamination, respectively. The gamma-ray hormesis in neutron irradiations was further confirmed by Ng et al. [27] who studied the neutron dose response on zebrafish embryos in vivo from 0.6 to 100 mGy with 14% of gamma-ray contamination. Ng et al. [27] demonstrated a suppression of the biological effect of neutrons when the associated gamma-ray contamination reached 10 mGy or above. On the other hand, Scott [28] and Scott et al. [29] investigated the induction of lung cancer on Wistar rats after they inhaled ^239^Pu (an alpha-particle emitter) in an insoluble dioxide form, with alpha-particle doses up to ~600 mGy. The authors showed that 1 to 2 mGy of gamma-ray doses suppressed lung-cancer induction in Wistar rats. The proposed mechanisms underlying the gamma-ray hormesis included removal of aberrant cells through apoptosis and high-fidelity DNA repair [30,31,32].

This phenomenon was generalized as “photon hormesis” in the present paper to include both X-ray and gamma-ray photons, in which the biological effect of a high-LET ionizing radiation was diminished by a low-LET photon exposure. Photon hormesis was also reported on suppressing the biological effect of alpha-particle radiation. For non-targeted ionizing effect, Ng et al. [33] reported that the onset of photon hormesis could mitigate the neutron-induced damages on zebrafish embryos and thus suppressed the induction of bystander signals to the neighboring naive embryos.

Despite the above interesting findings on interactions between high- and low-LET radiations, some results remained equivocal (e.g., [2,14,15,16,17,18,19,20]). Further studies on these interactions, particularly the underlying mechanisms, would be pertinent. In the present work, zebrafish (*Danio rerio*) embryos were employed as the in vivo vertebrate model to assess the effect of photon hormesis on dose responses to alpha particles. Zebrafish embryos had been commonly used in research related to toxicology screening and ionizing radiation [27,33,34,35,36,37,38,39,40,41,42,43,44,45,46,47,48,49,50] considering their numerous advantages, including their rapid development process, high fecundity, optically transparency and, most importantly, their genomes sharing considerable homology with human genomes [51]. It was established that different alpha-particle doses would lead to hormetic and toxic effects in zebrafish embryos [49], so a dose response curve for graded alpha-particle dose was established in the present work to fully assess the effect of photon hormesis. In relation, for the study on the effect of photon hormesis on neutron doses in zebrafish embryos, Ng et al. [27] examined the neutron dose response curve using graded neutron doses. For the study on the interaction between X-rays and alpha particles in lung epithelial cells, Brooks et al. [20] also used graded X-ray doses together with two alpha-particle doses. Furthermore, Brooks et al. [20] commented that time gaps between high- and low-LET exposures could weaken their interactions, so we also studied the effect of the time gap between alpha-particle and X-ray irradiations in the present work. Finally, an attempt was made to provide information on the mechanisms underlying the photon hormesis by studying the apoptotic counts at different time points after alpha-particle irradiations with and without additional X-ray irradiation.

## 2. Results

### 2.1. Part A: Effect of Alpha-Particle Dose with or without Additional Photon Dose

The dose responses of zebrafish embryos to different alpha-particle doses were first studied. Four alpha-particle doses, namely, 1.1, 2.2, 4.4, and 8.8 mGy, were employed in the present study. For each set of experiment, a total of 50 dechorionated embryos were deployed, which were divided into five groups each having 10 embryos, namely:

(1) *A_1_* group: embryos received ~1.1 mGy alpha-particle irradiation at 5 hpf;
(2) *A_2_* group: embryos received ~2.2 mGy alpha-particle irradiation at 5 hpf;
(3) *A_4_* group: embryos received ~4.4 mGy alpha-particle irradiation at 5 hpf;
(4) *A_8_* group: embryos received ~8.8 mGy alpha-particle irradiation at 5 hpf; and
(5) *A_C_* group: embryos were sham irradiated with alpha particles at 5 hpf.

To further study the effect of photon hormesis (from X-ray photons in the current study) on the dose response of alpha-particle irradiated zebrafish embryos, an additional X-ray dose of 10 mGy was delivered to the embryos immediately after their alpha-particle irradiation.

For each set of experiment in this section, a total of 50 dechorionated embryos were deployed, which were divided into five groups each having 10 embryos, namely:

(1) *AX_1_* group: embryos received ~1.1 mGy alpha-particle irradiation and 10 mGy X-ray irradiation at 5 hpf;
(2) *AX_2_* group: embryos received ~2.2 mGy alpha-particle irradiation and 10 mGy X-ray irradiation at 5 hpf;
(3) *AX_4_* group: embryos received ~4.4 mGy alpha-particle irradiation and 10 mGy X-ray irradiation at 5 hpf;
(4) *AX_8_* group: embryos received ~8.8 mGy alpha-particle irradiation and 10 mGy X-ray irradiation at 5 hpf; and
(5) *AX_C_* group: embryos were sham irradiated with alpha particles and X-rays at 5 hpf.

**Figure 1:**
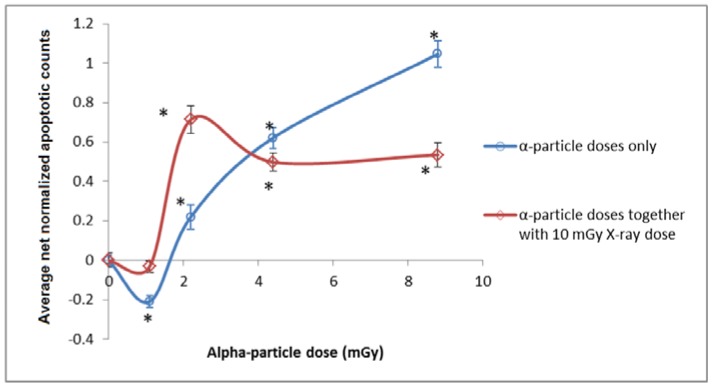
Net normalized apoptotic counts: (i) *N_AY_*^+^ and *N_Ac_*^+^, (ii) *N_AXY_*^+^ and *N_AXc_*^+^ on 24-hpf zebrafish embryos which have been irradiated with (i) alpha-particle dose only, and (ii) alpha-particle dose + 10 mGy X-ray at 5 hpf. Error bars represent the standard errors. The lines joining the data points are drawn to guide the eye only. Comparisons are separately made for the five data points within case (i) or case (ii) through ANOVA. When *p* ≤ 0.05 from ANOVA, post-hoc *t*-tests are further performed to assess the difference between tested *A_Y_* or *AX_Y_* groups and the corresponding *A_C_* or *AX_C_* group. Significant differences from post-hoc *t*-tests are asterisked in Figure 1. * Statistically significant differences between the tested *A_Y_* or *AX_Y_* group and the corresponding *A_C_* or *AX_C_* group.

The mean apoptotic counts (*N*) for the *A_1_*, *A_2_*, *A_4_*, *A_8_,* and *A_C_* groups were denoted as *N_A1_*, *N_A2_*, *N_A4_*, *N_A8_*, and *N_Ac_*, respectively. The average <*N_Ac_*> was computed from the *N_Ac_* values in the corresponding set of experiment. The “net normalized apoptotic counts” for all of the *A_1_*, *A_2_*, *A_4_*, and *A_8_* groups were determined as *N_AY_*^+^ = [(*N_AY_* − <*N_Ac_*>)/<*N_Ac_*>], where *Y* could assume values of 1, 2, 4, or 8, which corresponded to exposures to alpha-particle doses of 1.1, 2.2, 4.4, and 8.8 mGy, respectively. In relation, *N_Ac_*^+^ = [(*N_Ac_* − <*N_Ac_*>)/<*N_Ac_*>]. Similarly, the “net normalized apoptotic counts” for all the *AX_1_*, *AX_2_*, *AX_4_*, *AX_8_* groups were determined as *N_AXY_*^+^ = [(*N_AXY_* − <*N_AXc_*>]/[<*N_AXc_*>], where <*N_AXc_*> was computed from the *N_AXc_* values in the corresponding set of experiment. In relation, *N_AXc_*^+^ = [(*N_AXc_* − <*N_AXc_*>)/<*N_AXc_*>]. The net normalized apoptotic counts were grouped for analysis, and the results were shown in Figure 1. The data shown in Figure 1 for (i) alpha-particle dose only, and (ii) alpha-particle dose + X-ray dose were separately analyzed to confirm whether a particular case (dose or a combination of doses) would lead to hormetic or toxic effects, or would lead to effects insignificantly different from the background. ANOVA gave *p* = 9.69 × 10^−39^ for (i) alpha-particle dose only and *p* = 1.43 × 10^−25^ for (ii) alpha-particle dose + X-ray dose. When *p* ≤ 0.05 from ANOVA, the responses corresponding to at least two cases were significantly different, and post-hoc *t*-tests would be performed to assess the statistical significance between the *A_Y_* groups and the *A_C_* group for (i) alpha-particle dose only, and between the *AX_Y_* groups and the *AX_C_* group for (ii) alpha-particle dose + X-ray dose. When *p* ≤ 0.05/10 (i.e., 0.005) from a particular post-hoc *t*-test, the difference between the tested *A_Y_* or *AX_Y_* group and the corresponding *A_C_* or *AX_C_* group was considered statistically significant, and was asterisked in Figure 1. As revealed in Figure 1, 5-hpf embryos irradiated with 1.1 mGy of alpha particles exhibited hormetic effect and were thus in the hormetic zone (HZ), while those irradiated with ≥2.2 mGy of alpha particles exhibited toxic effect and were, thus, in the toxic zone (TZ) at 24 hpf. Furthermore, 5-hpf embryos irradiated with (1.1 mGy of alpha particles + 10 mGy of X-rays) did not lead to results significantly different from the background signals (i.e., apoptotic counts on *AX_C_* groups of embryos), while those irradiated with (≥2.2 mGy of alpha particles + 10 mGy of X-rays) were in the TZ.

To elucidate the effect of the additional X-ray dose on the alpha-particle irradiated embryos, the net normalized data in the three repeated experiments were combined for both parts and compared. The presence of photon hormesis was indicated by *N_AY_*^+^ > *N_AXY_*^+^, while cases with *p* ≤ 0.05 (assessed through *t*-tests) were considered statistically significant. The results and interpretations were:

(i) *N_A1_*^+^ < *N_AX1_*^+^, photon hormesis was absent, *p* = 3.44 × 10^−5^
(ii) *N_A2_*^+^ < *N_AX2_*^+^, photon hormesis was absent, *p* = 6.75 × 10^−10^
(iii) *N_A4_*^+^ > *N_AX4_*^+^, photon hormesis was present, *p* = 0.0185
(iv) *N_A8_*^+^ > *N_AX8_*^+^, photon hormesis was present, *p* = 1.19 × 10^−8^

Apparently, an additional X-ray dose of 10 mGy significantly altered the dose response of embryos at 24 hpf. For alpha-particle doses ≤ 2.2 mGy, the additional X-ray dose led to more apoptotic cells at 24 hpf. Interestingly, the significant hormetic effect originally recorded for the alpha-particle dose of 1.1 mGy no longer existed. In contrast, for alpha-particle doses ≥ 4.4 mGy, the additional X-ray dose significantly reduced the number of apoptotic cells at 24 hpf, which proved the presence of photon hormesis (to diminish the biological effect of alpha particles).

### 2.2. Part B: Effect of Time Gap between Alpha-Particle and X-ray Irradiations

To study the effect of time gap between alpha-particle and photon irradiation on the photon hormesis, the responses to alpha particles (4.4 mGy) in zebrafish embryos upon their receiving an additional X-ray dose (10 mGy) with time gaps *T* = 0 (i.e., immediately), 10, 15, 30, or 60 (min) were studied. Alpha-particle irradiation was performed at 5 hpf. Alpha-particle dose of 4.4 mGy and additional X-ray dose of 10 mGy were employed. In this part of the experiment, the apoptotic counts were determined at the time *H* = 24 (hpf), in contrast to *H* = 20 and 30 (hpf) in Part C below. A total of 30 dechorionated embryos, which were divided into three groups each having 10 embryos, were deployed in each set of experiment. The three groups were referred to as (with *H* = 24 in this part):

(1) *AX_H(T)_* group: embryos were irradiated with 4.4 mGy alpha-particle dose and then received 10 mGy X-ray dose with *H* = 24 (hpf) and *T* = 0, 10, 15, 30, or 60 (min);
(2) *A_H(T)_* group: embryos were irradiated with 4.4 mGy alpha-particle dose and then sham irradiated with X-rays, with *H* = 24 (hpf) and *T* = 0, 10, 15, 30, or 60 (min); and
(3) *C_H(T)_* group: embryos were sham irradiated with alpha particles and X-rays, with *H* = 24 (hpf) and *T* = 0, 10, 15, 30, or 60 (min).

**Figure 2:**
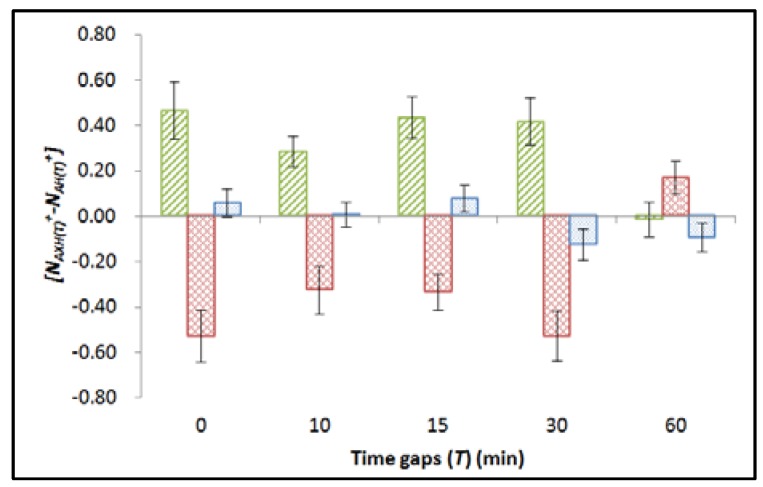
Differences between the net normalized apoptotic counts (*N_AXH(T)_*^+^ − *N_AH(T)_^+^*) on zebrafish embryos at 20 hpf (diagonally filled), 24 hpf (filled with diamond), and 30 hpf (filled with dots), which had been irradiated with an alpha-particle dose of 4.4 mGy at 5 hpf and an X-ray dose of 10 mGy, with *T* = 0, 10, 15, 30, or 60 (min) between the alpha-particle and X-ray irradiations. Error bars represent the standard errors. The error bars represent the standard errors.

The mean apoptotic counts (*N*) for the *AX_H(T)_*, *A_H(T)_* and *C_H(T)_* groups were denoted as *N_AXH(T)_*, *N_AH(T)_*, and *N_CH(T)_*, respectively. The average <*N_CH(T)_*> was computed from the *N_CH(T)_* values in the corresponding set of experiment. The “net normalized apoptotic counts” for the *AX_H(T)_*, *A_H(T)_* and *C_H(T)_* groups could be determined as *N_AXH(T)_*^+^ = [(*N_AXH(T)_* − <*N_CH(T)_*>)/<*N_CH(T)_*>], *N_AH(T)_*^+^ = [(*N_AH(T)_* − <*N_CH(T)_*>)/ <*N_CH(T)_*>] and *N_CH(T)_*^+^ = [(*N_CH(T)_* − <*N_CH(T)_*>)/<*N_CH(T)_*>], respectively. The net normalized apoptotic counts were grouped for analyses. The variations between all groups of embryos were first studied by ANOVA. For cases with *p* ≤ 0.05, post-hoc *t*-tests were then performed to assess the statistical significance between different groups. The results were summarized in Figure 2 and Table 1.

Apparently, photon hormesis was effective (i.e., *N_AX24(T)_*^+^ − *N_A24(T)_^+^* < 0) when *T* ≤ 30 min. Here the differences between the net normalized apoptotic counts (*N_AX24(T)_*^+^ − *N_A24(T)_^+^*) were largely independent of *T*. When *T* = 60 min, *N_AX24(T)_*^+^ > *N_A24(T)_^+^*, indicating that the additional X-ray dose could not induce photon hormesis but instead exacerbated the damages in the embryos.

### 2.3. Part C: Effect of Time Point at Which Apoptotic Counts Were Determined

To study the effect of the time point at which apoptotic counts were determined on the photon hormesis, the experiments in Part B above were repeated, but with *H* = 20 and 30 (hpf), in contrast to *H* = 24 (hpf) in Part B above. All embryos in the *AX_30(T)_*, *A_30(T)_* and *C_30(T)_* groups that would be analyzed at 30 hpf were treated with 75 µM 1-phenyl 2-thiourea (PTU) during embryogenesis at the 23.5 hpf (28 somite stage) as suggested by Karlsson et al. [52] to block the development of pigment cells within the embryos. A total of 30 dechorionated embryos, which were divided into three groups each having 10 embryos, were deployed in each set of experiment. The three groups were referred to as *AX_H(T)_*, *A_H(T)_* and *C_H(T)_* groups (with *H* = 20 and 30 in this part). The results for (*N_AXH(T)_*^+^ − *N_AH(T)_^+^*) for *H* = 20 and 30 were summarized in Figure 2 together with the results for *H* = 24 for comparison, while the results for *N_AX20(T)_^+^*, *N_A20(T)_^+^*, and *N_C20(T)_^+^* were also summarized in Table 2. The variations between all groups of embryos were first examined through ANOVA. For cases with *p* ≤ 0.05, the statistical significances between different groups were further investigated with post-hoc *t*-tests.

The results indicated that for cases with successful induction of photon hormesis observed at 24 hpf as shown in Part B (i.e., with *T* ≤ 30 (min)), all *N_AX20(T)_*^+^ were significantly larger than *N_A20(T)_*^+^ at 20 hpf. When *T* increased to 60 (min), where no photon hormesis was observed at 24 hpf, there was no significant difference between *N_AX20(T)_*^+^ and *N_A20(T)_*^+^. As regards the chosen time point of 30 hpf (25 h after alpha-particle irradiation), ANOVA among *N_AX30(T)_*^+^, *N_A30(T)_*^+^, and *N_C30(T)_*^+^ gave *p* values of 0.581, 0.874, 0.394, 0.150, and 0.371 for time gaps of 0, 10, 15, 30, and 60 min, respectively. Since *p* > 0.05 for all cases, no post-hoc *t*-tests were needed. In other words, the apoptotic counts of all irradiated groups of embryos had returned to the background level. The results are also shown in Figure 2.

## 3. Discussion

In the present study, we revealed that 5-hpf zebrafish embryos irradiated with 1.1 mGy of alpha particles displayed hormetic effects and were, thus, in the hormetic zone, while those irradiated with ≥2.2 mGy of alpha particles displayed toxic effects and were, thus, in the toxic zone when the apoptotic counts were determined at 24 hpf, which agreed with previous results [49]. It was also interesting to note that the hormetic effect induced by 1.1 mGy of alpha-particle irradiation disappeared when the zebrafish embryos were further irradiated with 10 mGy X-rays. Apparently, an additional X-ray dose of 10 mGy significantly altered the dose response of embryos at 24 hpf. In relation, Ng et al. [27] also demonstrated that photon hormesis was operative on zebrafish embryos for photon doses from 7 to 10 mGy. The present study showed that photon hormesis (with 10 mGy of X-rays) was operative only when the zebrafish embryos were irradiated with alpha particles with doses ≥ 4.4 mGy. The previously proposed mechanisms underlying the photon hormesis included high-fidelity DNA repair and removal of aberrant cells through apoptosis [30,31,32]. In the present study, when the embryos were irradiated with relatively small alpha-particle doses (≤2.2 mGy), the aggregate damages induced together with 10 mGy X-rays were not enough to trigger the high-fidelity DNA repair or removal of aberrant cells through apoptosis, and the additional X-ray dose would instead increase the apoptotic counts within the embryos. On the other hand, when an addition of 10 mGy X-rays was delivered to the embryos which had been irradiated with larger doses of alpha-particle radiation (i.e., 4.4 and 8.8 mGy), the aggregate damage was sufficiently large to induce an effective photon hormesis and, therefore, the apoptotic counts decreased.

In addition, when comparing embryos in the *AX_4_* and *AX_8_* groups, the apoptotic counts were similar (as shown by *p* = 0.48). This indicated that photon hormesis reduced the apoptotic counts to about the same level in embryos irradiated with alpha-particles doses above a certain threshold. Such observation agreed with the previous results of Ng et al. [27] who also demonstrated that when zebrafish embryos were irradiated with neutron doses of 70 and 100 mGy, where gamma-ray hormesis operated effectively, similar apoptotic counts remained. However, the reason behind these observations remained unclear and further studies would be needed to elucidate the underlying reasons.

As regards the effect of time gap between alpha-particle and X-ray irradiations, we demonstrated that photon hormesis was effective when the time gap was smaller than or equal to 30 min, but became ineffective when the time gap became 60 min. These results agreed with the assertion of Brooks et al. [20] that time gaps between high- and low-LET exposures could weaken their interactions, considering that sublethal damages produced by high-LET exposures were also rapidly repaired [53]. In particular, Ngo et al. [54] found that a time gap of 3 h between neon-ion and X-ray irradiations or between neutron and X-ray irradiations led to complete repair and, therefore, no interactions between the high- and low-LET exposures. In contrast, stronger interactions would occur for simultaneous exposures [20].

As regards the study on apoptotic counts determined at different time points, an interesting finding was revealed in that for cases with successful induction of photon hormesis observed at 24 hpf, all *N_AX20(T)_*^+^ were significantly larger than *N_A20(T)_*^+^ at 20 hpf. Furthermore, for these cases, (*N_AX20(T)_*^+^ − *N_A20(T)_^+^*) and (*N_AX24(T)_*^+^ − *N_A24(T)_^+^*) had comparable magnitudes but with opposite signs, and their trends were also commensurate with one another. Apparently, the drop in the apoptotic counts at 24 hpf was explained by bringing the apoptotic events earlier to 20 hpf. These results strongly supported the removal of aberrant cells through apoptosis as an underlying mechanism for photon hormesis. The same mechanism, i.e., removal of aberrant cells through apoptosis at an earlier time point also explained the hormetic effect in zebrafish embryos observed at 24 hpf upon their earlier exposure to a low concentration of uranium [46], in which case the apoptotic counts in the embryos also significantly increased 4 h before reaching 24 hpf.

Together with existing results in the literature, the present findings suggested that at least three conditions would be needed for photon hormesis to operate successfully: (1) the additional dosage of X-ray or gamma-ray photons should be sufficiently large, for example from 1–2 mGy and beyond on Wistar rats to act against alpha-particle irradiation [28,29], and between 7–10 mGy on zebrafish embryos to act against neutron irradiation [27]; (2) the damages inflicted by the primary ionizing radiation (which was the alpha-particle dose in the present study) should be above the threshold; and (3) the photon dose should be applied within a certain period of time after the primary dose.

The present results were obtained using apoptosis as the biological endpoint, which was also employed in previous related works on photon hormesis in zebrafish embryos [27,33,47,55] of which the present work was a continuation. Apoptosis was also used as the biological endpoint in many previous studies on radiobiological effects in zebrafish embryos (see e.g., [27,33,35,36,37,38,42,43,45,46,47,48,49]. As reviewed in [39], the extra apoptotic events developed in irradiated zebrafish embryos likely reflected genomic instability induced in the irradiated cells (at 5 hpf), since these irradiated cells and their earlier progeny, which were unstable and mutation prone, had appeared to be healthy and were able to survive many generations before the apoptosis pathways were initiated (at 20, 24, or 30 hpf). As such, it is anticipated that using other biological endpoints which characterize radiation-induced genomic instability or mutations will give qualitatively similar results.

## 4. Materials and Methods

### 4.1. Zebrafish Embryos

These animal studies in City University of Hong Kong were approved by the Department of Health, Government of the Hong Kong Special Administrative Region, with references [7,8,9,10,11,12,13] in DH/HA&P/8/2/5 Pt.1, and were performed in accordance with the guidelines.

Adult zebrafish (*Danio rerio*), both male and female, were mixed and maintained at 28.5 °C with a 14/10 h light-dark cycle. On the day of an experiment, a specially designed plastic collector was lowered into the fish tanks to collect embryos after the start of the 14-h photoperiod. To obtain a whole batch of embryos with the same developmental stages, the collection time for embryos was restricted to be no longer than 30 min. These collected embryos were then incubated in a 28.5 °C incubator immediately after collection, and were kept until they developed into 4 hpf for dechorionation. Healthy and well developed embryos were transferred into a new Petri dish with a thin agarose gel layer at the bottom and filled with E3 medium (5 mM NaCl, 0.33 mM MgSO_4_, 0.33 mM CaCl_2_, 0.17 mM KCl, and 0.1% methylene blue, ( Sigma-Aldrich, Co., St. Louis, MO, USA) for dechorionation under a stereomicroscope ( Nikon, Chi-yoda-ku, Tokyo, Japan). Since alpha particles had weak penetrating power, the chorion of each selected embryo was removed at 4 hpf to avoid the absorption of alpha-particle energy by the chorion and the enclosed embryonic fluid.

### 4.2. Alpha-Particle Irradiation

The setup for alpha-particle irradiation largely followed the design by Yum et al. [56]. Briefly, a 3.5 μm thick biocompatible Mylar film (Dupont, Hong Kong, China), which was glued to the bottom of a Petri dish having a 35 mm diameter hole, was used as the supporting substrate during the alpha-particle irradiation. In the present study, an ^241^Am alpha-particle source with alpha-particle energy of 5.49 MeV under vacuum and an activity of 4.26 kBq was employed.

### 4.3. X-Ray Irradiation

A self-contained X-ray irradiation system (X-RAD 320 irradiator, Precision X-Ray Inc., North Branford, CT, USA) with voltage and current set at 200 kVp and 2 mA, respectively, was employed to irradiate the zebrafish embryos. A 2.5 mm thick beam conditioning filter made of aluminum, copper and tin was employed. Under such a setting, the dose rate of irradiation was ~15 mGy/min. X-ray photons with the same characteristics were also employed in a previous study [47].

### 4.4. Experimental Protocols

#### 4.4.1. Part A: Effect of Alpha-Particle Dose with or without Additional Photon Dose

**Figure 3:**
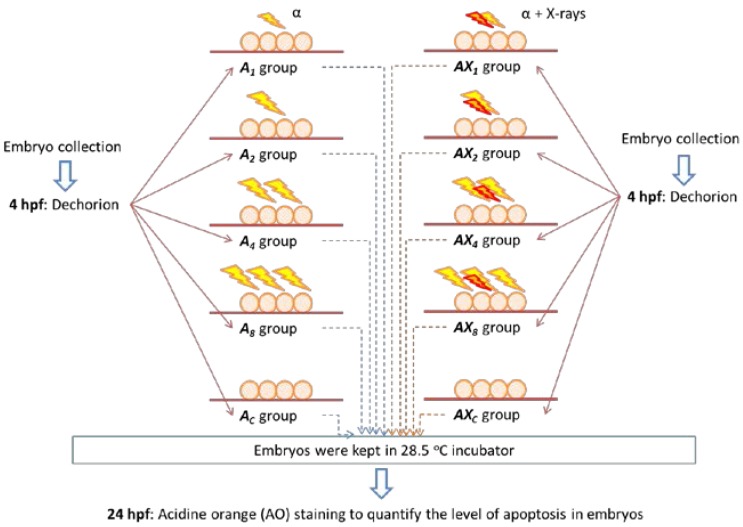
Schematic diagram showing the experimental steps involving embryos in the *A_1_*, *A_2_*, *A_4_*, *A_8_*, and *A_C_* groups, and *AX_1_*, *AX_2_*, *AX_4_*, *AX_8_*, and *AX_C_* groups.

To study the effect of photon hormesis on the dose responses of alpha particles in zebrafish, the dose responses of zebrafish embryos to different alpha-particle doses were first studied. As described in Section 2 above, in the first part of the experiments, embryos were divided into five groups, each having 10 embryos, namely: *A*_1_, *A*_2_, *A*_4_, and *A*_8_ groups which received ~1.1, ~2.2, ~4.4, and ~8.8 mGy alpha-particle irradiation at 5 hpf, and the *A_C_* group which was sham irradiated with alpha particles. In the second part of the experiment, an addition X-ray dose was delivered to the embryos immediately after their alpha-particle irradiation. As Ng et al. [27] showed that gamma-ray hormesis became effective in zebrafish embryos in reducing the apoptotic counts when the neutron dose was increased to above 50 mGy with 7 to 10 mGy of gamma ray contamination, a fixed X-ray dose of 10 mGy was employed in the present work to investigate the effect of photon hormesis. Embryos were divided into five groups each having 10 embryos, namely: *AX_1_*, *AX_2_*, *AX_4_*, and *AX_8_* groups which received ~1.1, ~2.2, ~4.4, and ~8.8 mGy alpha-particle irradiation together with 10 mGy X-ray irradiation at 5 hpf, and the *A_C_* group was sham irradiated with alpha particles and X-rays. A volume of 3 mL of E3 medium was used in these 10 agarose dishes and all embryos were incubated in an incubator at 28.5 °C until they reached 24 hpf. A total of three replicates of experiments were carried out independently. The experimental steps involving embryos in the *A_1_*, *A_2_*, *A_4_*, *A_8_*, and *A_C_* groups, and the *AX_1_*, *AX_2_*, *AX_4_*, *AX_8_*, and *AX_C_* groups are schematically shown in Figure 3.

#### 4.4.2. Part B: Effect of Time Gap between Alpha-Particle and X-ray Irradiations

**Figure 4:**
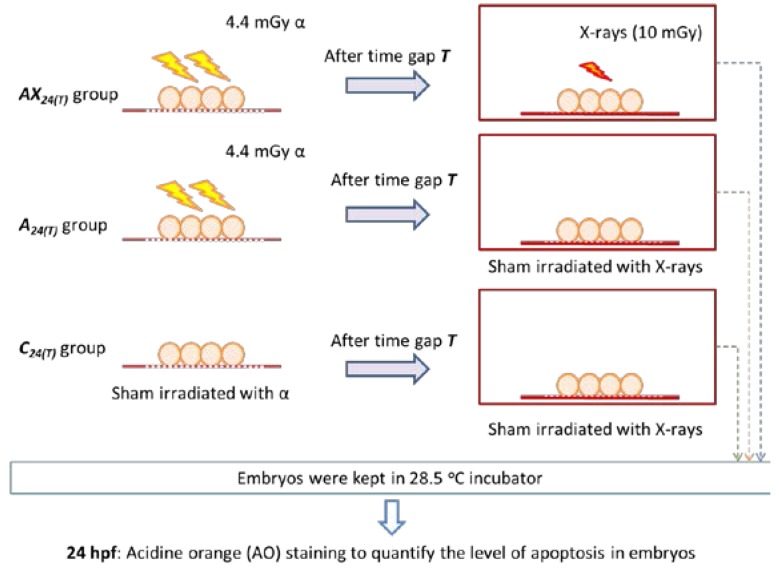
Schematic diagram showing the experimental steps involving embryos in the *AX_24(T)_*, *A_24(T)_*, and *C_24(T)_* groups, where time gaps *T* = 0, 10, 15, 30, or 60 (min) between alpha-particle and photon irradiations.

To study the effect of the time gap between alpha-particle and photon irradiation on the photon hormesis, the responses to alpha particles (4.4 mGy) in zebrafish embryos upon their receiving an additional X-ray dose (10 mGy) with time gaps *T* = 0, 10, 15, 30, or 60 (min) were studied. Alpha-particle irradiation was performed at 5 hpf. Alpha-particle dose of 4.4 mGy and additional X-ray dose of 10 mGy were employed in this part of the study. For each time gap, a total of three replicates of experiments were carried out independently. A total of 30 dechorionated embryos, which were divided into three groups each having 10 embryos, were deployed in each set of experiment. As described in Section 2 above, embryos were divided into 3 groups each having 10 embryos, namely: (a) *AX_24(T)_*, (b) *A_24(T)_*, and (c) *C_24(T)_* groups which were (a) irradiated with 4.4 mGy alpha-particle dose and then received 10 mGy X-ray dose with a time gap *T* = 0, 10, 15, 30, or 60 (min), (b) irradiated with 4.4 mGy alpha-particle dose and sham irradiated with X-rays with a time gap *T*, and (c) sham irradiated with alpha particles and X-rays with a time gap *T*, respectively. A volume of 3 mL of E3 medium was used in these three agarose dishes and all embryos were incubated in an incubator at 28.5 °C until they reached 24 hpf. The presence of photon hormesis could be revealed by comparing *N_AX24(T)_* and *N_A24(T)_* at 24 hpf. Figure 4 gives the schematic diagram of the experimental steps involving embryos in the *AX_24(T)_*, *A_24(T)_* and *C_24(T)_* groups.

#### 4.4.3. Part C: Effect of Time Point at Which Apoptotic Counts Were Determined

High-fidelity DNA repair and removal of aberrant cells through apoptosis were two proposed mechanisms for photon hormesis. Here, an attempt was made to discern between them through examining the apoptotic counts at different time points after alpha-particle irradiations with and without additional X-ray irradiation. Our data demonstrated that the effect of alpha-particle irradiation (4.4 mGy) on the induction of apoptotic cells diminished to a negligible level 25 h after the irradiation (i.e., at 30 hpf since alpha-particle irradiation was performed at 5 hpf) when compared with the sham-irradiated embryos. As such, in the present work, 20 hpf (which was 15 h after alpha-particle irradiation) and 30 hpf (which was 25 h after alpha-particle irradiation) were chosen as the two other time points at which apoptotic counts were determined. The experiments in Part B above were repeated, but with *H* = 20 and 30 (hpf), in contrast to *H* = 24 (hpf) in Part B above.

As zebrafish embryos begin to develop pigmentation, which constitute a prominent feature of the embryos, at around 25 hpf at 28.5 °C [57,58], these pigment cells might block the observation of the stained apoptotic cells. Thus, all embryos in the *AX_30(T)_*, *A_30(T)_* and *C_30(T)_* groups that would be analyzed at 30 hpf were treated with 75 µM 1-phenyl 2-thiourea (PTU) during embryogenesis at the 23.5 hpf (28 somite stage) as suggested by Karlsson et al. [52] to block the development of pigment cells within embryos. Treating zebrafish embryos at such a concentration of PTU (i.e., 75 µM) at 28 somite stage (i.e., 23.5 hpf) was effective in the generation of transparency without interfering the normal embryonic development of zebrafish embryos [52] and the level of apoptosis [46].

A total of 30 dechorionated embryos, which were divided into three groups each having 10 embryos, were deployed in each set of experiment. The three groups were referred to as *AX_H(T)_*, *A_H(T)_* and *C_H(T)_* groups (with *H* = 20 and 30 in this part). A volume of 3 mL of E3 medium was used in these three agarose dishes and all embryos were incubated at 28.5 °C in an incubator until they reached 20 or 30 hpf. For each time point at which the apoptotic counts were determined, a total of three sets of experiments were carried out independently. The variations between all groups of embryos were first examined through ANOVA. For cases with *p* ≤ 0.05, the statistical significances between different groups were further investigated with post-hoc *t*-tests.

### 4.5. Quantification of Apoptosis by Vital Dye Staining

Apoptosis was chosen as the biological endpoint in the present study. When the embryos developed into 24 (for Parts A and B), 20, or 30 hpf (for Part C), they were transferred into a medium with 2 μg/mL of a vital dye, acridine orange (AO). Embryos were kept in the staining solution in the dark for 60 min, and then washed twice in the culture medium to remove the excess dye. The embryos were anaesthetized using 0.0016 M tricaine (Sigma, St. Louis, MO, USA) before examining under the fluorescent microscope. For each embryo, three images focusing on different sections of the embryo were captured with a magnification of 40×. The images were then combined into a single image for quantification of the apoptotic cells. A computer program “Particle Counting 2.0” (developed by J. Zhang) was employed for apoptotic counts in each embryo.

### 4.6. Data Analysis

In each part of the experiments (i.e., Parts A, B, and C, as described above), three replicates of experiments were performed independently on different days. The apoptotic counts on each whole embryo were determined as described above. The differences between different groups were assessed first by studying the variations between all the groups of embryos in that part of study by ANOVA. Cases with *p* values ≤ 0.05 were considered to correspond to statistically significant differences between at least two of the compared groups. In such cases, post-hoc *t*-tests were then performed to assess the statistical significance between different groups.

## 5. Conclusions

The present paper examined the effect of X-ray-induced photon hormesis on dose responses to alpha particles in zebrafish embryos, through the revelation of apoptotic events by acridine orange (AO) staining. Photon hormesis was observed at 24 hpf for an X-ray dose of 10 mGy if the alpha-particle dose was ≥4.4 mGy, but not for smaller alpha-particle doses, probably due to insufficient inflicted aggregate damages. Photon hormesis was induced if the time gaps *T* were ≤30 min between the delivery of the alpha-particle dose (4.4 mGy) and the X-ray dose (10 mGy), but was absent when *T* = 60 min, at which time repair of damage induced by alpha particles would have completed to prevent their interactions with those induced by X-rays. The reduced apoptotic counts recorded at 24 hpf in the presence of photon hormesis was explained by bringing the apoptotic events earlier to 20 hpf, which supported the removal of aberrant cells through apoptosis as an underlying mechanism for photon hormesis.

## Figures and Tables

**Table 1:** ANOVA and post-hoc t-test results obtained among *N_AX24(T)_*^+^, *N_A24(T)_*^+^, and *N_C24(T)_*^+^, with time gaps *T* = 0, 10, 15, 30, or 60 (min) between the alpha-particle and X-ray irradiations, and with apoptotic counts determined at *H* = 24 (hpf).

*T* (min)	ANOVA ^a^	Post-Hoc *t*-Tests		Photon Hormesis
		*N_AX24(T)_*^+^ vs. *N_C24(T)_*^+^^b^	*N_AX24(T)_*^+^ vs. *N_A24(T)_*^+^^c^	
0	*p* = 1.02 × 10^−11^ *	*p* = 1.37 × 10^−6^ **	*p* = 2.58 × 10^−5^ **	Yes (*N_AX24(0)_*^+^ < *N_A24(0)_*^+^)
10	*p* = 6.74 × 10^−11^ *	*p* = 1.17 × 10^−5^ **	*p* = 1.50 × 10^−3^ **	Yes (*N_AX24(10)_^+^ < N_A24(10)_^+^*)
15	*p* = 2.34 × 10^−13^ *	*p* = 3.10 × 10^−6^ **	*p* = 4.40 × 10^−5^ **	Yes (*N_AX24(15)_^+^ < N_A24(15)_^+^*)
30	*p* = 4.38 × 10^−17^ *	*p* = 1.28 × 10^−7^ **	*p* = 6.04 × 10^−6^ **	Yes (*N_AX24(30)_^+^ < N_A24(30)_^+^*)
60	*p* = 1.71 × 10^−12^ *	*p* = 1.09 × 10^−10^ **	*p* = 0.0113 **	No (*N_AX24(60)_^+^ > N_A24(60)_^+^*)

^a^ Comparison among *N_AX24(T)_*^+^, *N_A24(T)_*^+^, and *N_C24(T)_*^+^ using ANOVA. Post-hoc *t*-tests were performed for cases with *p* ≤ 0.05, which were considered statistically significant and asterisked; ^b^ *p* values obtained in post-hoc *t*-tests performed between *N_AX24(T)_*^+^ and *N_C24(T)_*^+^. Cases with *p* ≤ 0.0167 (i.e., 0.05/3) were considered statistically significant and double-asterisked; ^c^ *p* values obtained in post-hoc *t*-tests performed between *N_AX24(T)_*^+^ and *N_A24(T)_*^+^. Cases with *p* ≤ 0.0167 (i.e., 0.05/3) were considered statistically significant and double-asterisked.

**Table 2:** ANOVA and post-hoc *t*-test results obtained among *N_AX20(T)_*^+^, *N_A20(T)_*^+^, and *N_C20(T)_*^+^, with time gaps *T* = 0, 10, 15, 30, or 60 (min) between the alpha-particle and X-ray irradiations, and with apoptotic counts determined at *H* = 20 (hpf).

*T* (min)	ANOVA ^a^	Post-Hoc *t*-Tests		Photon Hormesis
		*N_AX20(T)_*^+^ vs. *N_C20(T)_*^+^^b^	*N_AX20(T)_*^+^ vs. *N_A20(T)_*^+^^c^	
0	*p* = 1.21 × 10^−15^ *	*p* = 1.04 × 10^−11^ **	*p* = 2.94 × 10^−4^ **	No (*N_AX20(0)_^+^ > N_A20(0)_^+^*)
10	*p* = 3.22 × 10^−15^ *	*p* = 2.40 × 10^−13^ **	*p* = 6.49 × 10^−5^ **	No (*N_AX20(10)_^+^ > N_A20(10)_^+^*)
15	*p* = 9.23 × 10^−17^ *	*p* = 9.88 × 10^−15^ **	*p* = 5.01 × 10^−6^ **	No (*N_AX20(15)_^+^ > N_A20(15)_^+^*)
30	*p* = 5.58 × 10^−9^ *	*p* = 8.26 × 10^−9^ **	*p* = 1.00 × 10^−4^ **	No (*N_AX20(30)_^+^ > N_A20(30)_^+^*)
60	*p* = 2.98 × 10^−6^ *	*p* = 2.28 × 10^−7^ **	*p* = 0.414	No (*N_AX20(60)_^+^ ~ N_A20(60)_^+^*)

^a^ Comparison among *N_AX20(T)_*^+^, *N_A20(T)_*^+^, and *N_C20(T)_*^+^ using ANOVA. Post-hoc *t*-tests were performed for cases with *p* ≤ 0.05, which were considered statistically significant and asterisked; ^b^ *p* values obtained in post-hoc *t*-tests performed between *N_AX20(T)_*^+^ and *N_C20(T)_*^+^. Cases with *p* ≤ 0.0167 (i.e., 0.05/3) were considered statistically significant and double-asterisked; ^c^ *p* values obtained in post-hoc *t*-tests performed between *N_AX20(T)_*^+^ and *N_A20(T)_*^+^. Cases with *p* ≤ 0.0167 (i.e., 0.05/3) were considered statistically significant and double-asterisked.
